# Supplementary material for: Engineering circular RNA for potent and stable translation in eukaryotic cells
Source: Nat Commun. 2018 Jul 6;9:2629. doi: 10.1038/s41467-018-05096-6 (PMC6035260; doi:10.1038/s41467-018-05096-6)
Supplement: Supplementary file 1 — Supplementary Information [file 41467_2018_5096_MOESM1_ESM.pdf]

Engineering Circular RNA for Potent and Stable Translation in Eukaryotic Cells

Wesselhoeft et al.

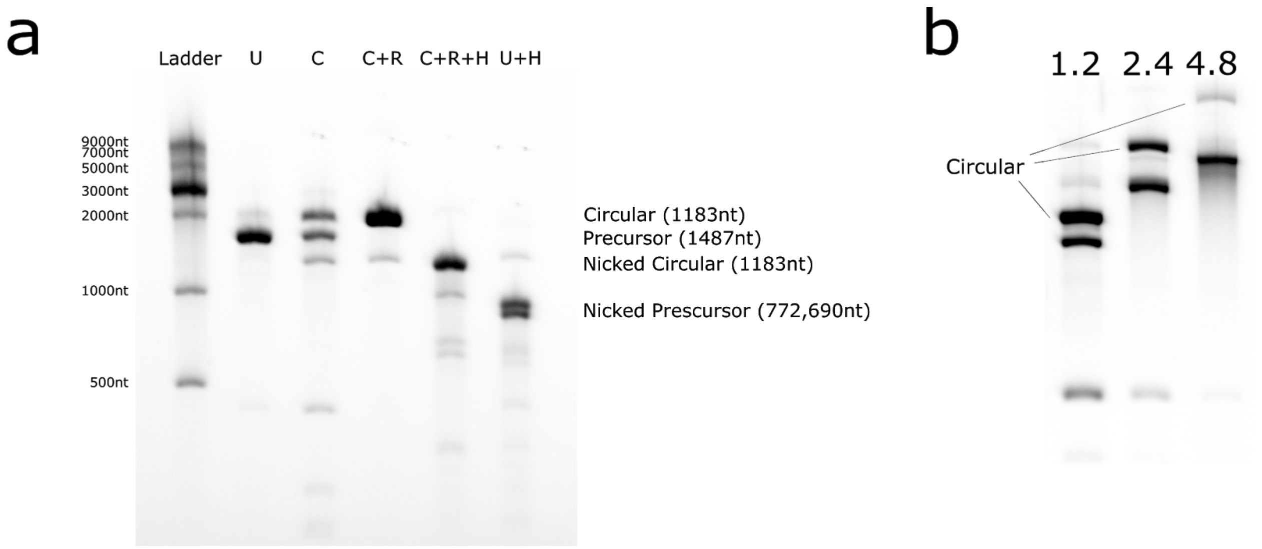

Supplementary Figure 1. a) Agarose gel of different RNA species in splicing reactions and RNAse H-treated samples. U: precursor RNA not subjected to circularization conditions. C: precursor RNA (with strong homology arms) subjected to circularization conditions. C+R: Lane C, digested with RNase R. C+R+H: Lane C+R, digested with oligonucleotide-guided RNase H. U+H: Lane U, digested with oligonucleotide-guided RNase H. **b)** The effect of insert length on RNA circularization efficiency using a permuted self-splicing group I intron containing optimized spacers and homology arms. 1.2: 1200nt circRNA. 2.4: 2400nt circRNA. 4.8: 4800nt circRNA.

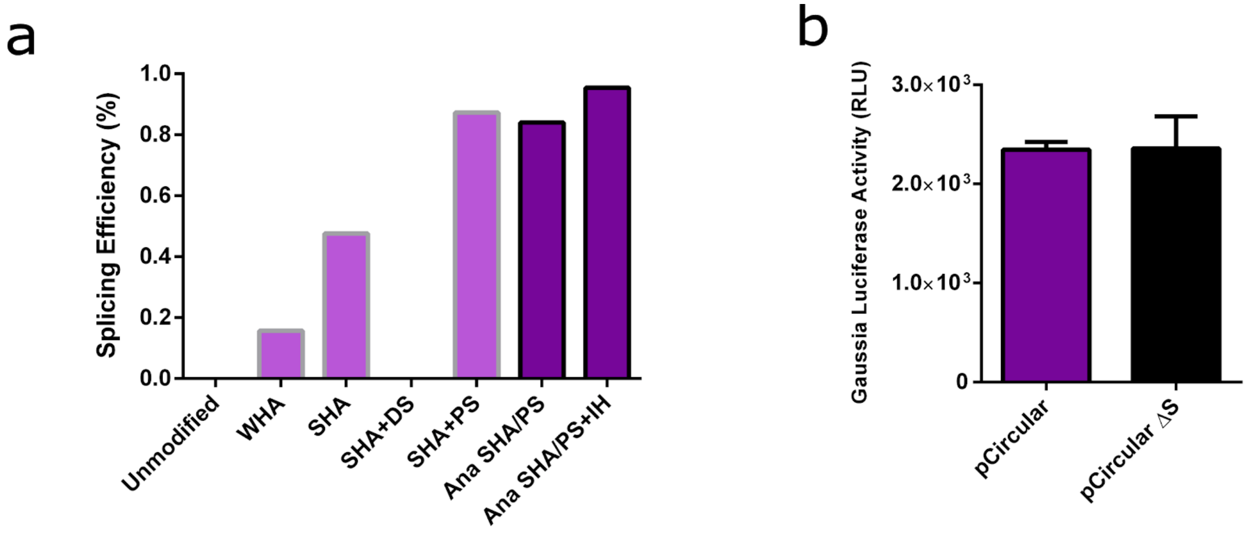

Supplementary Figure 2. a) Gel quantification of splicing efficiency of precursor molecules containing different engineered sequences. WHA: weak homology arms. SHA: strong homology arms. DS: disruptive spacer. PS: permissive spacer. Ana: Anabaena base PIE sequences. IH: internal homology. **b)** Splicing-optimized precursor sequences (Fig. 3a) were placed into a plasmid under the control of a CMV promoter (pCircular) and GLuc activity was compared between this construct and the same construct with deleted splice sites (pCircular ΔS) at 24 hours post transfection in HEK293 cells. No significant differences were observed between the two conditions, suggesting that transcripts resulting from self-splicing plasmids do not display enhanced stability, consistent with the possibility that PIE-based constructs do not post-transcriptionally circularize in mammalian cells (data presented as mean+SD, n=3).

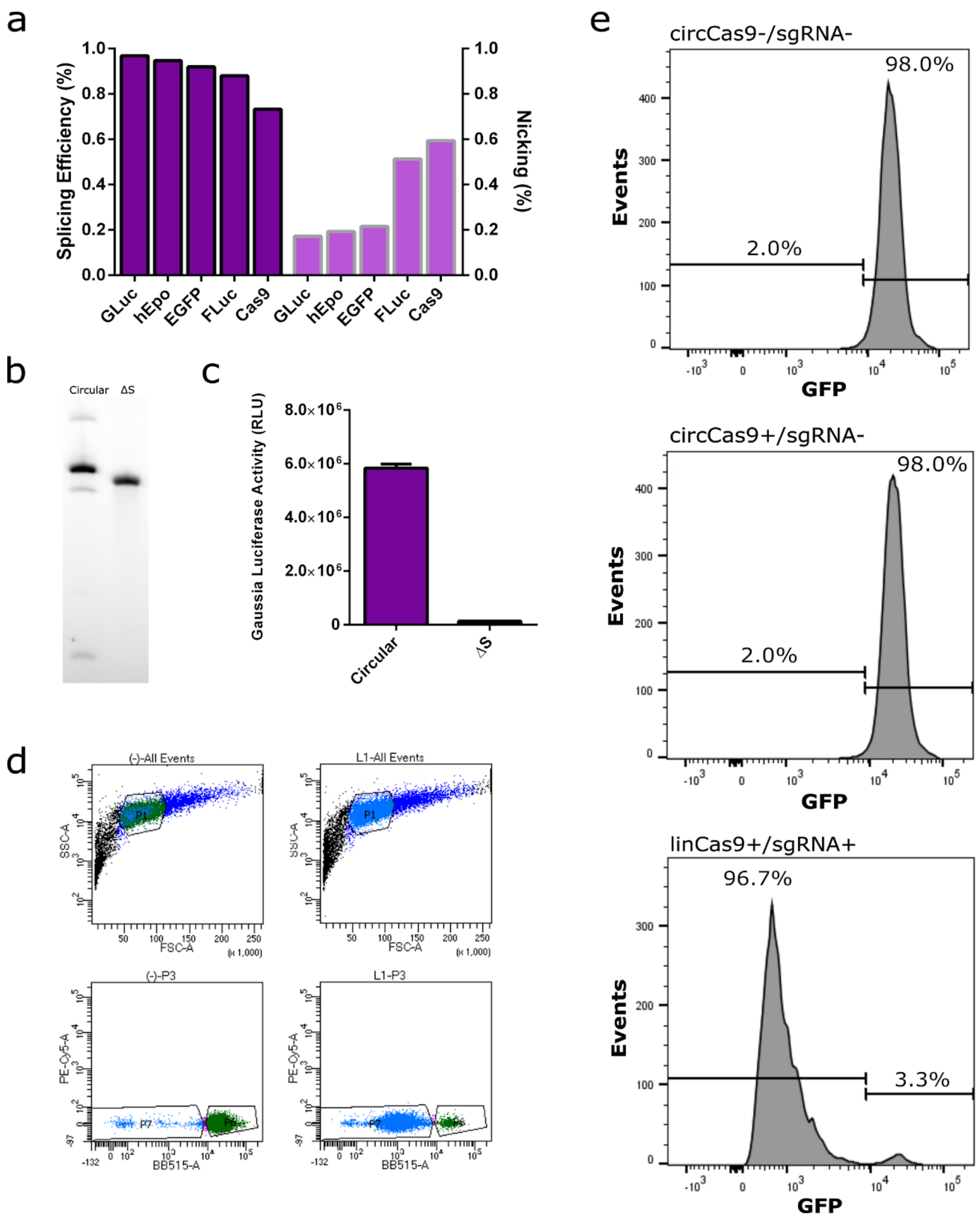

Supplementary Figure 3. a) Gel quantification (ImageJ) of splicing efficiency and nicking in circRNA containing different intervening coding regions, arranged by length. Splicing efficiency presented as ratio of non-precursor (circular, nicked) to precursor RNA. Nicking presented as ratio of nicked RNA to non-nicked long RNA (precursor, circular). **b)** Agarose gel demonstrating the effect of small deletions encompassing the 5’ and 3’ splice sites on splicing. **c)** Luminescence in the supernatant of HEK293 cells 24 hours after transfection with circRNA coding for GLuc and containing an EMCV IRES or the same precursor RNA with deleted splice sites (data presented as mean+SD, n=4). **d)** Gating strategies for analysis of the data presented in Fig. 3h. Left: circCas9-. Right: circCas9+. **e)** Additional controls for the data shown in Fig. 3h: GFP fluorescence in HEK293-EF1a-GFP cells without transfection (top) or 4 days after transfection with circRNA coding for Cas9 alone (circCas9+/sgRNA-, middle) or linear mRNA coding for Cas9 cotransfected with sgGFP (linCas9+/sgRNA+, bottom).

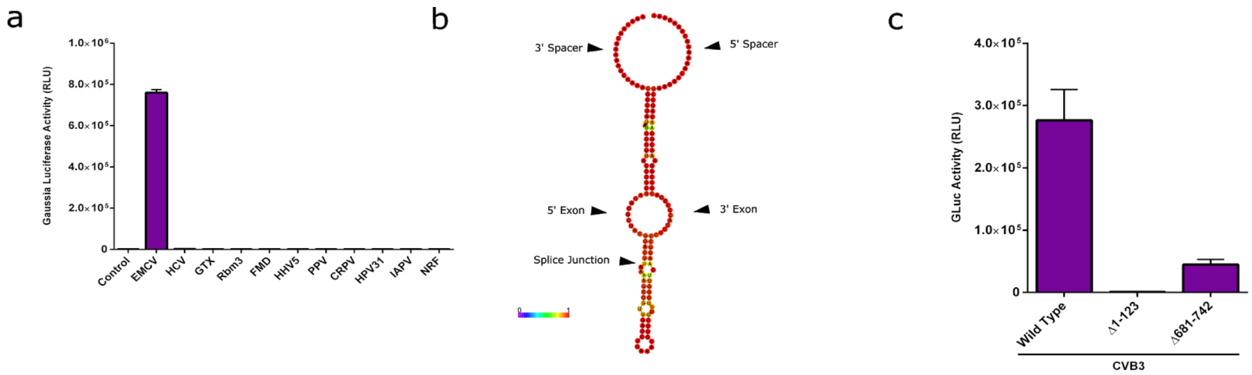

Supplementary Figure 4. a) Additional IRES sequences and putative IRES sequences tested for functionality in the context of circRNA (n=3). **b)** RNAFold prediction of precursor RNA secondary structure at the splice junction. IRES, coding region, and introns are excluded. **c)** Luminescence in the supernatant of HEK293 cells 24 hours after transfection with circRNA containing the wild type CVB3 IRES, the CVB3 IRES with a proximal deletion (Δ1-123), or the CVB3 IRES with a distal deletion (Δ681-742) (n=4) (all data presented as mean+SD).

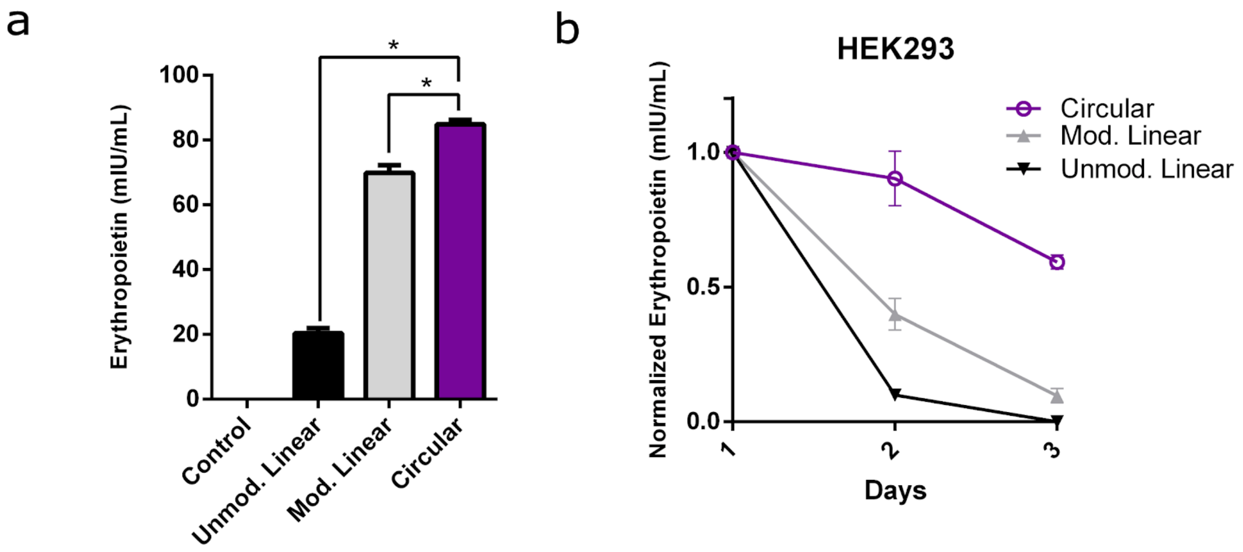

Supplementary Figure 5. a) Human erythropoietin in the supernatant of HEK293 cells 24 hours after transfection with CVB3-hEpo-pAC circRNA or 5-methoxyuridine-modified or unmodified linear GLuc mRNA. **b)** Human erythropoietin in the supernatant of HEK293 cells starting 24 hours after transfection with CVB3-hEpo-pAC circRNA or 5-methoxyuridine-modified or unmodified linear hEpo mRNA and continuing for 3 days (all data data presented as mean+SD, n=4, *p<0.05 (Welch's t-test)).

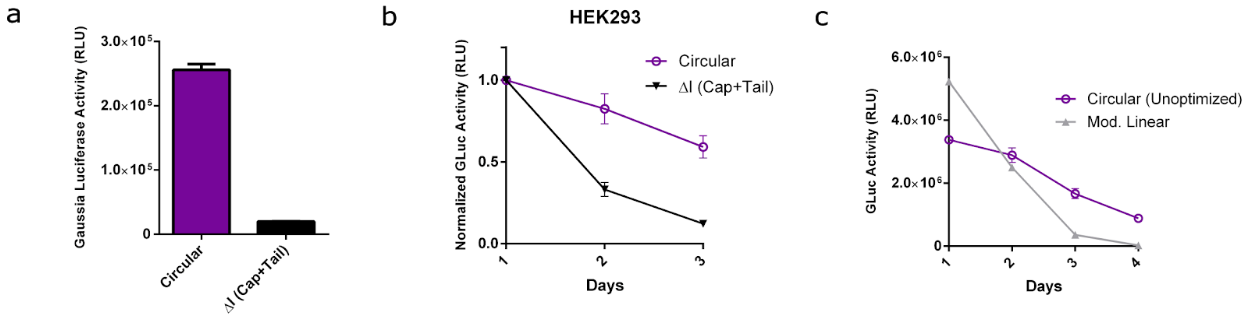

Supplementary Figure 6. a) Luminescence in the supernatant of HEK293 cells 24 hours after transfection with circRNA coding for GLuc and containing a CVB3 IRES or the same precursor RNA with deleted introns (ΔI), a 5' methylguanosine cap, and a 3' polyA tail. **b)** Luminescence in the supernatant of HEK293 cells starting 24 hours after transfection with CVB3-GLuc-pAC circRNA or ΔI-CVB3-GLuc-pAC linear mRNA and continuing for 3 days. **c)** Luminescence in the supernatant of HEK293 cells starting 24 hours after equal weight transfection with unoptimized GLuc circRNA or modified linear GLuc mRNA and continuing for 4 days (all data presented as mean+SD, n=3).

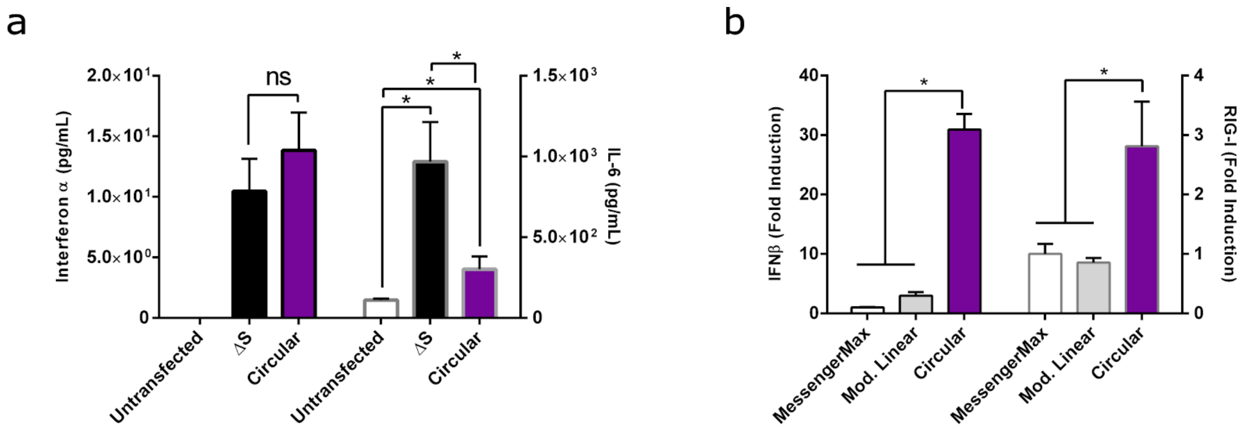

Supplementary Figure 7. a) IFN-α and IL-6 detected in the supernatant of A549 cells 24 hours after transfection with 40ng of HPLC-purified, RNase R enriched circRNA coding for GLuc and containing a CVB3 IRES or the same HPLC-purified precursor RNA with deleted splice sites (ΔS), a 5' methylguanosine cap, and a 3' polyA tail (n=3). **b)** IFN-β and RIG-I transcript mean fold induction 24 hours after equal weight transfection of HeLa cells with GLuc circRNA or modified linear GLuc mRNA, assessed by qPCR (all data presented as mean+SD, n=3, ns=not significant, *p<0.05 (Welch's t-test)).

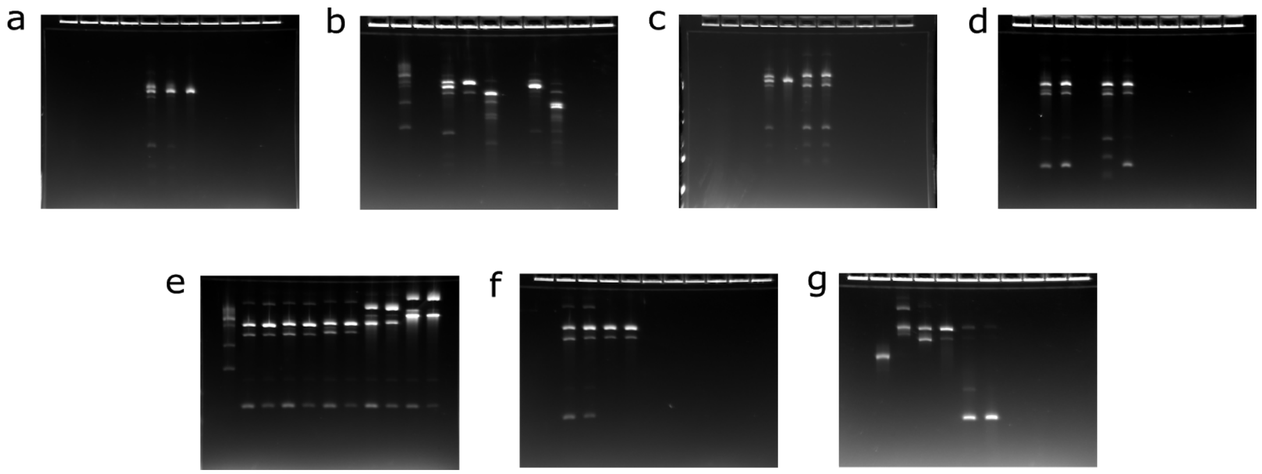

Supplementary Figure 8. Unprocessed gel images. **a)** Unprocessed image from Fig. 1c. Note that the final image is a mirror image of the unprocessed image. **b)** Unprocessed image from Fig. 1d, including ladder (first from left). **c)** Unprocessed image from Fig. 2b. **d)** Unprocessed image from Figs. 2d (left pair) and 2e (right pair). **e)** Unprocessed image from Fig. 3a, including ladder (first from left). **f)** Unprocessed image from Fig. 5b. **g)** Unprocessed image from Fig. 5a, including linear mRNA (first from left).

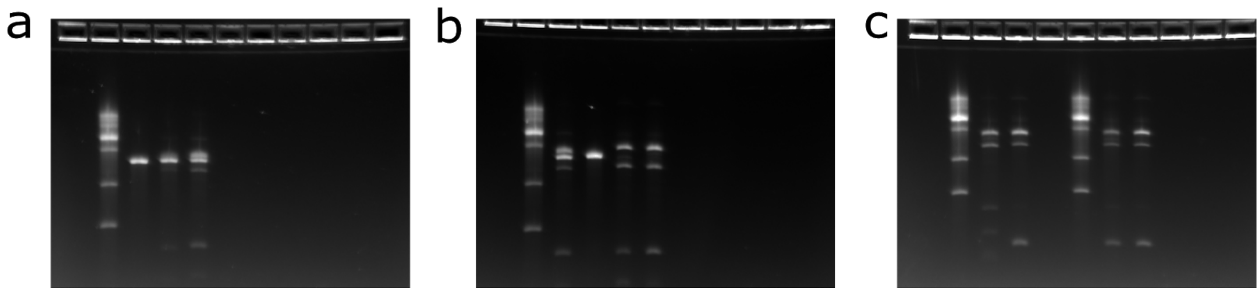

Supplementary Figure 9. Gel images with ladders. See main text for description of non-ladder lanes. **a)** Unprocessed gel image of samples from Fig. 1c, with ladder (first from left). **b)** Unprocessed gel image of samples from Fig. 2b, with ladder (first from left). **c)** Unprocessed gel image of samples from Fig. 2e (left three) and Fig. 2d (right three) with ladder (first from left for each triplet).
