# Supplementary material for: Engineering circular RNA for potent and stable translation in eukaryotic cells
Source: Nat Commun. 2018 Jul 6;9:2629. doi: 10.1038/s41467-018-05096-6 (PMC6035260; doi:10.1038/s41467-018-05096-6)
Supplement: Supplementary file 3 — Description of Additional Supplementary Files [file 41467_2018_5096_MOESM3_ESM.pdf]

Description of Additional Supplementary Files

File Name: Supplementary Data 1
Description: Sequences used in this study.
